# GDF15 Is Elevated in Eating Disorders and Is Involved in the Gut‐Brain Axis via Ghrelin

**DOI:** 10.1002/erv.70018

**Published:** 2025-07-24

**Authors:** Livio Tarchi, Giulia Brancolini, Sara Giachetti, Gaia Maiolini, Emanuele Cassioli, Eleonora Rossi, Gianluca Villa, Rachele Garella, Roberta Squecco, Paolo Rovero, Paolo Comeglio, Valdo Ricca, Francesco De Logu, Romina Nassini, Giovanni Castellini

**Affiliations:** ^1^ Department of Health Sciences, Center for Integrated Translational Research and Knowledge Transfer (INCLINE) University of Florence Florence Italy; ^2^ Department of Experimental and Clinical Medicine University of Florence Florence Italy; ^3^ Department of Neuroscience, Psychology, Pharmacology and Infant Health University of Florence Florence Italy; ^4^ Department of Experimental, Clinical, and Biomedical Sciences “Mario Serio” University of Florence Florence Italy

**Keywords:** appetite control, metabolic regulation, MIC‐1, neuroendocrine signalling, weight dysregulation

## Abstract

**Objective:**

GDF15 induces anorexia and visceral discomfort, regulating appetite, food intake and potentially metabolic responses. However, its role in eating disorders remains unexplored.

**Method:**

A total of 145 participants were recruited (60 patients with anorexia nervosa, 20 with bulimia nervosa, 13 with binge eating disorder, 52 participants from the general population). Ghrelin and GDF15 serum levels were measured with immunosorbent assay kits.

**Results:**

Ghrelin was elevated in patients with an eating disorder compared to healthy controls (age and BMI‐adjusted ANCOVA, *F*‐value 4.084, *p*‐value 0.008). GDF15 was significantly correlated with ghrelin (Spearman rho 0.430, *p*‐value < 0.001) and BMI (rho = −0.176, *p*‐value = 0.025). GDF15 predicted the BMI of patients with anorexia nervosa and individuals from the general population, again being elevated at lower BMI (linear regression beta −0.254, *p*‐value 0.005). The effect of GDF15 on BMI was observed as under the mediation of ghrelin (direct effect −0.056, *p*‐value 0.577; indirect effect −0.199, *p*‐value < 0.001).

**Conclusions:**

Present results provide novel insights into the role of GDF15 in eating disorders, describing its serum level in this clinical population for the first time. In addition, a positive correlation between GDF15 and ghrelin serum levels is also reported for the first time.

## Summary

- This study is the first to report serum GDF15 levels in patients with an eating disorder.
- GDF15 was elevated in patients with an eating disorder.
- In both patients and controls, GDF15 was correlated with ghrelin.

## 1 Introduction

Growth Differentiation Factor 15 (GDF15) is a protein gaining significant attention in recent years (Cimino and Coll 2022), given its role in regulating eating behaviours (Klein et al. 2021) and, possibly, metabolic pathways (Aguilar‐Recarte et al. 2021). GDF15 was first known as Macrophage Inhibitory Cytokine‐1 (MIC‐1), and was first described as a member of the transforming growth factor‐β (TGF‐β) superfamily (Bootcov et al. 1997). However, after its receptor became better characterised (i.e. GFRAL; Mullican et al. 2017), GDF15 has emerged as structurally and functionally pertaining to Glial cell line‐Derived Neurotrophic Factors (GDNF).

GDF15 is produced by multiple tissues in the body, including the liver, heart, and adipose tissue, and it is especially secreted in response to cellular stress or inflammation (Tarchi et al. 2025). GDF15 has recently emerged as inducing anorexia and visceral malaise, as evaluated in animal models (Borner et al. 2020) and patients with cancer (Buchholz et al. 2021). Recent research has also highlighted a protective metabolic effect of GDF15 against high‐fat diets (Takeuchi et al. 2024). However, GDF15 has been described as elevated both in under and overweight patients (Igual‐Gil et al. 2024; Keipert and Ost 2021; Molfino et al. 2020), as well as in obesity (Keipert and Ost 2021; Tran et al. 2018) and in patients with diabetes (Xu et al. 2022).

Different hypotheses emerged regarding this dual role of GDF15 in regulating food intake and body weight. To date, a debate continues whether GDF15 induces anorexia and weight loss solely activating central receptors (Hes et al. 2025; Hsu et al. 2017), or by engaging in a wider network of gut‐brain regulation factors (Lockhart et al. 2020; Wang et al. 2024). In fact, more recent evidence has characterised GFRAL as not only restricted to the hindbrain, but widely expressed across different peripheral tissues (Fichtner et al. 2024; Garella et al. 2025), mediating gut‐brain crosstalk (Garella et al. 2025; Igual‐Gil et al. 2024; Tsai et al. 2019), and leveraging leptin pathways (Breit et al. 2023).

The presence of peripheral receptors might help explaining this above‐mentioned double role of GDF15 on food intake. In fact, elevations in GDF15 have been observed at least as a complementary mechanism not only for weight loss, but also restored glycaemic control, as evaluated for pharmacological interventions not typically conceptualized as exerting a central effect, including artesunate (Guo et al. 2024), camptothecin (J. F. Lu et al. 2022) and metformin (Coll et al. 2020). As previously mentioned, GDF15 has been shown to influence appetite and food intake, inducing anorexia and visceral malaise. Emerging evidence however described GDF15 not only as influencing food intake, but also food preferences. In fact, GDF15 elevation, beyond the previously mentioned metabolic effect, was also associated with preferentially reduced fat consumption in animal models (Tran et al. 2018). Nonetheless, the role of GDF15 in eating disorders (ED), which are characterised by food restriction, selective food preference, and possibly gut‐brain dysregulation (Achamrah et al. 2019; Fairburn et al. 2008; Rossi et al. 2021), remains mostly unexplored.

In EDs, recent research has focused on the potential identification of diagnostic and prognostic biomarkers (Dani et al. 2024; Peters et al. 2021), as pharmacological therapy still remains limited (Bulik 2021). The identification and validation of specific molecular targets may stimulate empirically‐informed pharmacological developments, possibly aimed at addressing the neglected needs of patients with EDs. Therefore, the characterisation of GDF15 in EDs seems of primary interest. Previous evidence may also aid in better characterising ED from a biological perspective. In fact, previous research has described ghrelin as increased across all main EDs, namely Anorexia Nervosa (AN), Bulimia Nervosa (BN) and Binge Eating Disorder (BED) (Atalayer et al. 2012; Seidel et al. 2021). Yet, the role of ghrelin in EDs may be further elucidated in light of its interplay with other potential biomarkers.

However, to better understand the relationship between ghrelin and other potential biomarkers, potential confounders need to be acknowledged. Indeed, the elevation of ghrelin in EDs is complicated by the exposure to major childhood traumatic events, which are often reported in this clinical population (Rossi et al. 2021). In brief, ghrelin may increase after exposure to major childhood traumatic events (Rossi et al. 2021), and its increase in ED may in turn underlie increased loss of control over food intake in BN or BED (Atalayer et al. 2012), but also food restriction in AN (Rossi et al. 2021). Interestingly, molecular studies suggested that GDF15 may stimulate ghrelin secretion (Wittekind et al. 2021). More specifically, ghrelin plasma levels have been associated with GFRAL expression, as evaluated by a transcriptome‐wide association study (Wittekind et al. 2021), suggesting that GDF15 may induce ghrelin secretion (Müller et al. 2015). However, the interplay between GDF15 and ghrelin remains to be determined, both in the general population and in patients with ED, although both hormones have emerged as key regulators of food intake.

To be noted, previous reports on GDF15's serum levels, across a variety of conditions, may need to be carefully re‐assessed. Indeed, a significant number of previous reports employed enzyme‐linked immunosorbent assay (ELISA) known to underreport GDF15 (Karusheva et al. 2022). In fact, a common genetic variant (histidine to aspartate at position 202 in the pro‐peptide) has been described as amongst the targets of common immunoassays (Karusheva et al. 2022). The current study thus aims to further characterise GDF15 in individuals from the general population and in patients diagnosed with ED (AN, BN and BED), while acknowledging the need for specific ELISA kits to properly determine GDF15 levels in human participants. Moreover, the current study aims to explore the association between GDF15 and ghrelin in humans, and testing specific hypotheses on the relationship between these two regulators of food intake.

### 1.1 Aims

The primary aim of the current study was to assess and describe differences in GDF15 between patients with ED and individuals from the general population, mainly focussing on patients with AN. This diagnostic group is, in fact, the one with most limited pharmacological therapy options (Bulik 2021). The secondary aims were (1) to explore the potential association between GDF15 and ghrelin (positing a positive association), (2) to test different hypothesis regarding the potential relationship between GDF15, EDs, and ghrelin. More specifically, three different hypotheses were tested: (i) GDF15 discriminates between patients with AN and individuals from the general population, as GDF15 induces ghrelin increases, and thus gut‐brain axis dysregulation; (ii) GDF15 predicts BMI in patients with AN, being positively associated with psychological severity; (iii) GDF15 increases in patients with AN, as related to other known risk factors, such as exposure to major childhood traumatic experiences (Rossi et al. 2024).

## 2 Methods

### 2.1 Sample Enrolment

The present study was designed as observational and cross‐sectional. A consecutive series of female patients (first time seeking outpatient care) who attended the Eating Disorders Clinic of the University Hospital of Florence, Italy, was enroled between March 2018 and December 2024. Inclusion criteria were: female sex, female gender, age between 18 and 65 years, diagnosis of AN, BN or BED according to the Diagnostic and Statistical Manual of Mental Disorders, Fifth Edition (DSM‐5: American Psychiatric Association 2013), as assessed by the Structured Clinical Interview for DSM‐5 Disorders, Clinician Version (First et al. 2016). Exclusion criteria were as follows: illiteracy, intellectual disability, presence of psychotic symptoms, severe depression, or manic state at the time of enrolment, current suicidal ideation, and lack of written informed consent. Patients were also excluded if they were under current use of psychoactive medications, with the exception of antidepressant medication and benzodiazepines, unless they were changed in the last month.

For individuals from the general population, inclusion criteria included: female sex, female gender, age between 18 and 65 years, no previous or current diagnosis of AN, BN or BED according to the Diagnostic and Statistical Manual of Mental Disorders, Fifth Edition (American Psychiatric Association 2013), as assessed by the Structured Clinical Interview for DSM‐5 Disorders, Clinician Version (First et al. 2016). Exclusion criteria for individuals from the general population were: illiteracy, intellectual disability, presence of psychotic symptoms, severe depression, or manic state at the time of enrolment, current suicidal ideation, presence of compensatory behaviours (vomiting, inappropriate drug use, excessive and inappropriate physical exercise as a form of weight and shape control), and lack of written informed consent.

### 2.2 Instruments and Data Collection

During the structured clinical interview (First et al. 2016), clinicians recorded exposure to major childhood traumatic experiences (sexual abuse, emotional abuse, physical abuse or neglect). After structured clinical interviews, clinicians recorded the following socio‐demographic data: age and sex. On the first day of admission to the clinic, patients were evaluated for their anthropometric measures from which BMI was calculated. Blood withdrawal was conducted on the same day, while fasting for at least 12 h, in the morning. Furthermore, participants were also asked to complete the following validated psychometric questionnaires:

- Symptom Checklist‐90‐Revised (SCL‐90), a 90‐item self‐reported symptom inventory to assess psychological symptoms and mental distress. It is composed of a global score (Global Severity Index, GSI) to measure overall psychological distress level. Higher scores indicate more severe psychological distress. The Cronbach's alpha for each domain in the Italian translation ranges between 0.70 and 0.96 (Prunas et al. 2012). SCL GSI ranges from 0 to 4.
- Beck Depression Inventory II (BDI), a 21‐item self‐reported questionnaire to assess depressive symptoms. The BDI can be either conceptualised as composed of a bifactorial structure (mental and physical symptoms) or rather a unidimensional, higher‐order global score (Sica and Ghisi 2007). Higher scores indicate more severe depressive symptoms. The Cronbach's alpha for its global score has been reported as 0.840 (Maggi et al. 2023). BDI ranges from 0 to 63.
- Eating Disorder Examination Questionnaire 6.0 (EDE), a 28‐item self‐reported questionnaire to evaluate the range, frequency, and severity of behaviours associated with a diagnosis of ED. EDE provides a global score (EDE Total Score) as well as an indication of the burden of compensatory behaviours (EDE Vomiting; EDE Laxative use; EDE Physical Exercise). Higher scores correspond to higher levels of psychopathology. The Cronbach's alpha for each subscale in the Italian translation ranges between 0.79 and 0.94 (Calugi et al. 2017). EDE Total Score ranges from 0 to 6, while compensatory behaviours are expressed as number of days with symptoms within a timeframe of 28 days—thus ranging from 0 to 28.

### 2.3 Ghrelin and GDF15

As previously mentioned, blood collection was performed on the same day of admission to the clinic for eating disorders for patients, in the morning while fasting for at least 12 h. Psychometric assessments were also performed, in the morning, while fasting for at least 12 h. Whole blood was then centrifuged (2000 × g for 10 min at 4°C) and plasma was collected. Serum ghrelin was measured using a single‐analyte ELISA kit (#BMS2192, Thermo Fisher Scientific, Waltham, MA). Data are expressed as ng/mL. GDF15 levels were also measured using a single‐analyte ELISA kit (#ab155432, Abcam, Cambridge, UK) according to the manufacturer's protocol. Protein concentration was also determined using the Bradford assay. Data are expressed as pg/mg of protein.

### 2.4 Ethics Approval and Informed Consent

All subjects provided informed consent for study participation. The study protocol was approved by the Ethics Committee of the Institution of the promoting centre (IRB codes OSS.14.162 and OSS.24.354, last update to the ethical committee approved in November 2023).

### 2.5 Statistical Analysis

Descriptive statistics were first computed, by means and standard deviations. Group differences for age and BMI were estimated by analysis of variance (ANOVA). Group differences for ghrelin, GDF15 and psychometrics (SCL‐90 GSI, BDI, EDE Total Score, EDE Vomiting, EDE Laxative Use, EDE Physical Exercise) were computed by analysis of covariance (ANCOVA), adjusting for both age and BMI. Post‐hoc differences for each diagnostic contrast were then assessed, after correction for multiple comparisons (Bonferroni). As GDF15 was not normally distributed (Shapiro‐Wilk test *p* < 0.001) control analyses were conducted to assess group differences with non‐parametric tests (Kruskal–Wallis). Factors associated with GDF15 were explored by Spearman's rho coefficients.

Further analyses were conducted comparing patients with AN and individuals from the general population. First, Mann‐Whitney U tests were computed to estimate the group difference for patients with AN diagnosed with either restrictive or binge‐purging subtype. Then, heterogeneity was explored, by means of standard deviation and by testing equality of variances between subgroups (Levene's test). Group differences were then tested for diagnostic subtypes by ANCOVA, controlling for the confounding effect of age, BMI and ghrelin.

The diagnostic potential to discriminate between patients with AN and individuals from the general population was explored employing logistic regression, composed of an intercept, and two independent variables (ghrelin, GDF15). The diagnostic potential was assessed by computing sensitivity, specificity, area under the curve (AUC), and coefficient of determination (Nagelkerke 1991).

The potential of GDF15 in stratifying patients with AN was also explored in terms of severity (i.e. BMI thresholds, American Psychiatric Association 2013). For this purpose, ANOVA was adopted to compute group differences according to severity. Post‐hoc differences for each contrast were then assessed, after correction for multiple comparisons (Bonferroni).

First, linear regression was employed to assess the relationship between GDF15 and BMI (total effect). Then, the hypothesis that GDF15 is related to increases in ghrelin, and, in turn, these increases in ghrelin predict BMI was tested (i.e. the indirect effect of GDF15 > direct effect). As a control analysis, an inverted model was also estimated (BMI predicting GDF15 under the mediation of ghrelin).

Second, linear regression was employed to estimate the interaction term between GDF15 and psychological severity (EDE Total Score). To estimate the increase in variance explained, model comparison was computed, using a comparator with a base model predicting BMI only by age and EDE total score.

Third, linear regression was employed to estimate the interaction term between GDF15 and exposure to major childhood traumatic experiences. For this purpose, a dummy variable was employed (1 if the patient was exposed to major childhood traumatic experiences, 0 if not). To estimate the increase in variance explained, model comparison was computed, using a comparator with a base model without interaction terms, predicting BMI only by age and exposure to childhood traumatic experiences.

## 3 Results

A total of 145 participants were recruited for the study. Ghrelin was elevated in AN in comparison to HC (mean difference = 4.496, Bonferroni corrected *p*‐value < 0.001, Cohen's *d* = 1.83). As GDF15 was not normally distributed, group differences were also assessed by the Kruskal‐Wallis test (overall effect of diagnostic groups, *p*‐value = 0.007). This test confirmed that patients with AN and BN exhibited increased GDF15 in comparison to individuals from the general population (AN contrast: *p*‐value = 0.014 and BN contrast: *p*‐value = 0.005—Bonferroni corrected). See Table 1 for further details.

**TABLE 1:** Descriptive statistics.

	Anorexia nervosa (*n* = 60)	Bulimia nervosa (*n* = 20)	Binge eating disorder (*n* = 13)	General population (*n* = 52)	*F*‐value (age and BMI‐adjusted ANCOVA)
Age^a^	24.96 ± 11.31	24.09 ± 7.72	33.36 ± 9.83	25.40 ± 3.20	2.896*
BMI^a^	16.46 ± 1.94	22.50 ± 8.18	36.46 ± 8.63	21.51 ± 1.48	78.820***
Ghrelin	8.50 ± 5.76	6.00 ± 3.52	2.50 ± 0.96	3.89 ± 1.86	3.040*
GDF15	205.14 ± 120.53	190.19 ± 127.29	196.18 ± 118.04	185.66 ± 96.59	4.084**
SCL90 GSI	1.33 ± 0.79	1.91 ± 0.70	1.52 ± 0.73	0.54 ± 0.29	10.078***
BDI	23.19 ± 12.70	24.56 ± 10.84	25.50 ± 6.99	7.09 ± 3.98	10.412***
EDE total score	3.07 ± 1.60	3.37 ± 1.61	3.46 ± 1.72	0.49 ± 0.30	14.425***
EDE vomiting	1.58 ± 4.84	3.04 ± 6.16	2.00 ± 6.33	—	0.685
EDE laxative use	0.80 ± 3.50	1.28 ± 2.62	0.00 ± 0.00	—	0.492
EDE physical exercise	3.78 ± 7.61	1.46 ± 2.59	1.11 ± 3.33	—	1.166

*Note:* As individuals from the general population were excluded if compensatory behaviours were reported, EDE Vomiting, Laxative Use and Physical Exercise (invariably reported as 0.00 ± 0.00), group differences were assessed only within patients.

Abbreviations: ANCOVA = analysis of covariance; ± Standard Deviation, BDI = Beck Depression Inventory, EDE = Eating Disorder Examination Questionnaire, GSI = Global Severity Index, SCL90 = Symptom Checklist 90 Revised.

^a^ Analysis of variance—not adjusted ANOVA.

* *p* < 0.05.

** *p* < 0.01.

*** *p* < 0.001.

**FIGURE 1:**
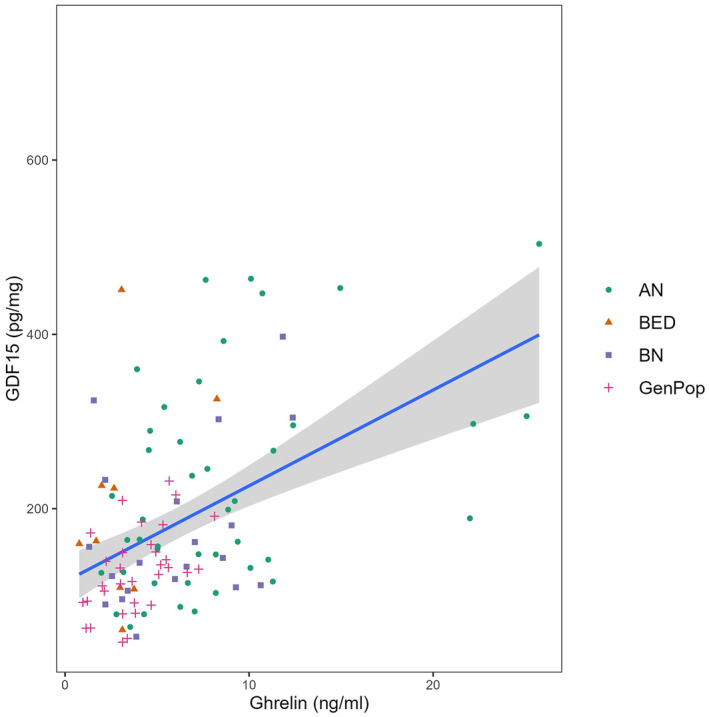
GDF15 is highly and positively correlated with ghrelin (Spearman rho 0.430, *p* < 0.001). Here, a scatter plot is colour coded by diagnosis. Linear regression is visually represented (beta = 10.990, *p* < 0.001). AN = patients with Anorexia Nervosa, BN = patients with Bulimia Nervosa, BED = patients with Binge Eating Disorder, GenPop = individuals from the general population.

GDF15 was highly and positively correlated with ghrelin (rho = 0.430, *p*‐value < 0.001, see Figure 1), while moderately and negatively correlated with BMI (rho = −0.176, *p*‐value = 0.025). GDF15 serum levels were not correlated with age (rho = 0.079, *p*‐value = 0.420). Similarly, GDF15 was not significantly correlated with psychological distress (SCL90 GSI: rho = −0.121, *p*‐value = 0.183), depressive symptoms (BDI rho = −0.018, *p*‐value = 0.848), overall eating psychopathology (EDE Total Score: rho = −0.052, *p*‐value = 0.545), nor compensatory behaviours (vomiting: rho = −0.038, *p*‐value = 0.670; laxative use: rho = −0.030, *p*‐value = 0.741; physical exercise: rho = −0.123, *p*‐value = 0.181). GDF15 was highly and positively correlated with ghrelin even when restricting analyses to subsamples (patients with AN: rho = 0.417, *p*‐value = 0.005, individuals from the general population: rho = 0.483, *p*‐value = 0.005).

### 3.1 GDF15 in AN, Subtypes

Further analyses were conducted comparing patients with AN (AN‐r, 38 patients out of 60%–63.33%; AN‐bp, 22 patients out of 60%–36.67%) and individuals from the general population. The diagnostic potential of GDF15 was then explored, assessing the potential to discriminate between patients with AN and individuals from the general population. Analyses showed moderate sensitivity and specificity (0.750 and 0.767, respectively) for a diagnostic algorithm employing both ghrelin and GDF15 serum levels (AUC = 0.839, Nagelkerke *R* ^2^ = 0.462).

Ghrelin and GDF15 were not different between diagnostic subtypes in patients with AN (ghrelin: 8.59 ± 6.98 for AN‐r and 8.35 ± 5.77 for AN‐bp, Mann‐Whitney *p*‐value = 0.987; GDF15 188.50 ± 107.018 for AN‐r and 233.89 ± 147.78 for AN‐bp, Mann‐Whitney *p*‐value = 0.393). However, GDF15 exhibited high heterogeneity and a significant difference in variances between the two subtypes (Levene's test, *F*‐value = 5.298, *p*‐value = 0.025). Group differences were then tested for diagnostic subtypes controlling for the confounding effect of age, BMI and ghrelin. Results showed increased GDF15 in AN‐bp in comparison to AN‐r, adjusting for age, BMI and ghrelin (marginal mean 277.31 vs. 186.63, *p*‐value = 0.038). Subsequent analyses, adjusting for additional variables, showed that only EDE Physical Exercise was significantly associated with GDF15 serum levels (EDE Physical Exercise: *F*‐value = 4.726, *p*‐value = 0.033; subtype difference: *F*‐value = 13.539, *p*‐value = < 0.001). Exposure to major childhood traumatic did not significantly influence observed differences between subtypes (main effect: *F*‐value = 0.058, *p*‐value = 0.811; subtype difference: *F*‐value = 4.326, *p*‐value = 0.042; interaction effect: *F* = 1.417, *p*‐value = 0.239).

### 3.2 Diagnostic Severity

**FIGURE 2:**
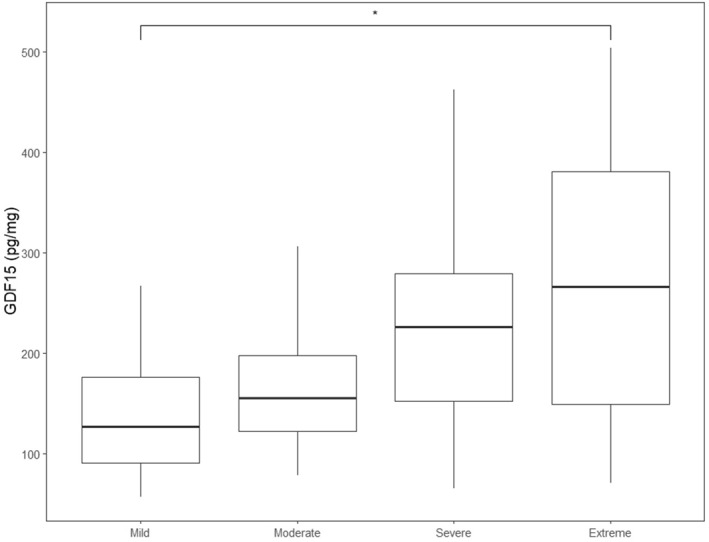
GDF15 discriminates patients with AN according to diagnostic severity (i.e., BMI thresholds, as described within DSM‐5 and DSM‐5‐TR). Post‐hoc group differences revealed a significant contrast between patients with mild and extreme Anorexia Nervosa (AN): mean difference +101.81 in extreme patients, *p* < 0.041 Bonferroni corrected.

The potential to stratify patients according to severity was then explored (patients with AN: mild, 27 patients out of 60%–45.00%; moderate 6 patients out of 60%–10.00%, severe 12 patients out of 60%–20.00%, extreme 15 patients out of 60%–25.00%). GDF15 followed a gradient of severity and was significantly different according to severity (*F*‐value = 2.779, *p*‐value = 0.049). Post‐hoc group differences revealed a significant difference for patients with either extreme or mild AN (see Figure 2).

### 3.3 GDF15 and Ghrelin

**FIGURE 3:**
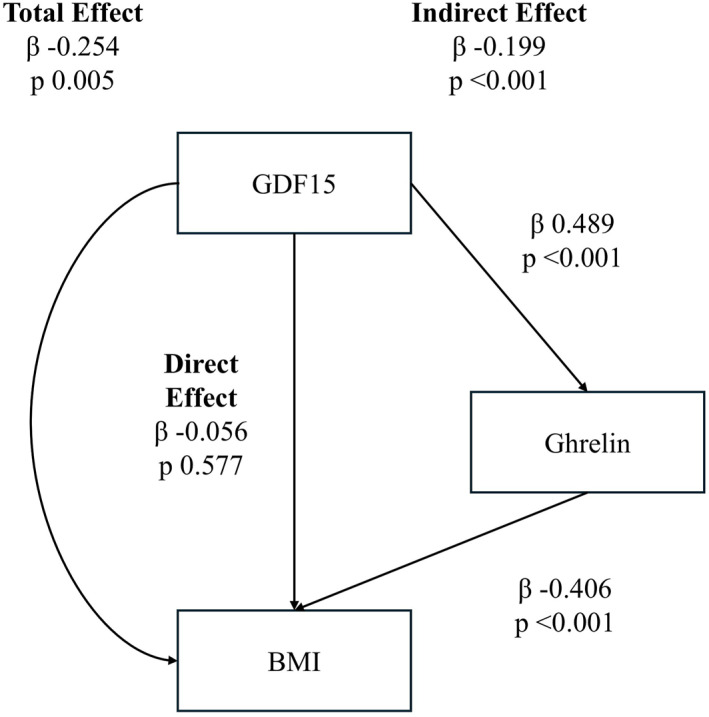
GDF15 predicts BMI in patients with AN and individuals from the general population (total effect), being elevated at lower BMI. However, the direct effect of GDF15 was not significantly predictive of BMI. On the other hand, the indirect effect of GDF15 on BMI by ghrelin was significant (indirect effect).

GDF15's potential to induce gut‐brain axis dysregulation was then assessed by mediation analysis. GDF15 was able to predict the BMI of patients with AN and individuals from the general population, being elevated at lower BMI (total effect, see Figure 3). However, in the full mediation model, the direct effect of GDF15 was not significantly predictive of BMI (direct effect, see Figure 3). On the other hand, the indirect effect of GDF15 on BMI was observed as mediated by an elevation in ghrelin (Figure 3). An inverted model was not supported (BMI predicting GDF15: beta = −0.052, *p*‐value = 0.580).

### 3.4 Psychological Correlates

**FIGURE 4:**
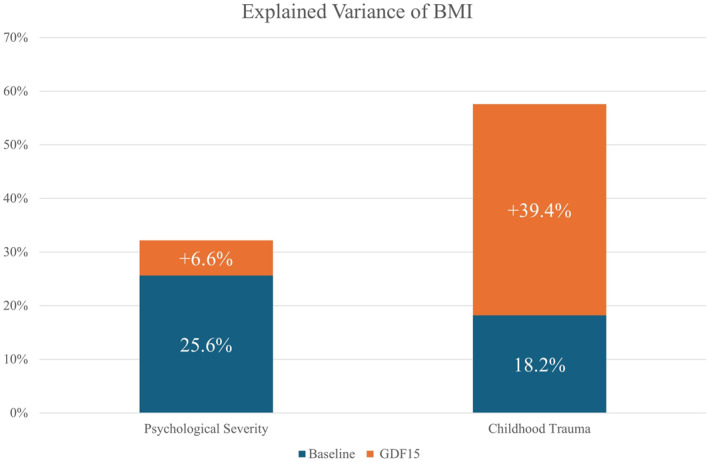
GDF15 better predicts BMI as a function of exposure to major childhood trauma. A linear model employing psychological severity (EDE Total Score) only accounted for 25.6% of BMI variance. Adding GDF15 serum levels and interaction effects did not significantly improve the prediction (+6.6%, *p* < 0.088). By contrast, exposure to major childhood trauma accounted for 18.2% of BMI variance, but adding GDF15 and interaction effects significantly improved the prediction (+39.4%, *p* < 0.001) reaching 57.6% of variance explained.

GDF15's potential to predict BMI could derive from increases in GDF15 serum levels as related to psychological severity. In other words, psychological severity may increase GDF15 serum levels, and the interaction between psychological severity and GDF15 may also thus predict BMI. The additional variance explained by GDF15 was not significantly increased, and amounted only to 6.6% (i.e. in comparison to a base model employing age and EDE Total Score; *p*‐value = 0.088, see Figure 4). To be noted GDF15 did not predict BMI directly (beta = 0.009, *p*‐value = 0.146), but rather as a function of EDE Total Score (interaction term: beta = −0.004, *p*‐value = 0.040).

### 3.5 Childhood Traumatic Experiences

GDF15's potential to predict BMI may derive from concurrent increases as related to known risk factors, as exposure to major childhood traumatic experiences. In other words, exposure to major childhood traumatic experiences may increase GDF15 serum levels, and the interaction between childhood traumatic experiences and GDF15 may thus in turn predict BMI. The additional variance explained by GDF15 was here significant and amounted to 35.3% (i.e. in comparison to a base model employing age and exposure to major childhood traumatic experiences; *p*‐value < 0.001, see Figure 4). To be noted GDF15 predicted BMI directly (beta = −0.011, *p*‐value = < 0.001), and by interacting with the exposure to major childhood traumatic experiences (interaction term: beta = 0.011, *p*‐value = 0.006).

## 4 Discussion

Present results provide novel insights into the role of GDF15 in EDs. First, the current study provides evidence for the first time of GDF15 serum levels in patients with ED. Results show that GDF15 may be described as elevated across EDs, especially in patients with AN. Second, present results show that GDF15 may stratify patients with AN according to diagnostic severity, suggesting a future potential as a diagnostic biomarker in this clinical population. GDF15 was positively associated with ghrelin, with significant implication for future translational studies on appetite and food intake regulation. Importantly, present results provide preliminary evidence for this translational potential, suggesting that the relationship between GDF15 and diagnostic severity in EDs is better explained as under the mediation of ghrelin serum levels.

As previously mentioned, current findings indicate that GDF15 levels are significantly elevated in patients with EDs compared to individuals from the general population. This aligns with previous research suggesting that GDF15 is a stress‐responsive cytokine involved in appetite regulation and metabolic adaptation (Cimino and Coll 2022; Keipert and Ost 2021). In the context of EDs, the upregulation of GDF15 may reflect an adaptive response to chronic caloric restriction, weight loss, or metabolic stress. Given its established role in inducing aversive feeding behaviour and nausea‐like symptoms, elevated GDF15 may contribute to the reduced food intake and altered appetite regulation observed in individuals with EDs.

Therefore, increased GDF15 serum levels in patients with ED may partly explain eating restriction across AN, BN and BED (American Psychiatric Association 2013), as well as the frequent report of visceral malaise in these clinical populations (Zucker and Bulik 2020). The positive association between GDF15 and ghrelin may also partly explain loss of control over food intake (i.e. bulimic symptoms), a common complaint even in most severe patients with AN (American Psychiatric Association 2013).

An important finding of this study is that GDF15 levels may serve as a biomarker for stratifying patients with AN based on diagnostic severity. Specifically, higher GDF15 levels were observed among more severe patients with AN, suggesting its potential as a diagnostic biomarker. The ability to stratify patients based on a biological marker could be valuable for tailoring treatment approaches and monitoring disease progression. In fact, GDF15 has consistently been described as predictive of higher cardiovascular risk in other clinical population, linking food intake, inflammation and cellular ageing (di Candia et al. 2021).

GDF15 was found to discriminate between AN subtypes, with greater levels in patients with AN‐bp in comparison to AN‐r, possibly due to increased compensatory exercise in this group. Current results also suggest that major childhood traumatic experiences are positively associated with GDF15 serum levels, although subtype differences between patients with AN‐bp in comparison to AN‐r were not primarily driven by this factor alone. This elevation of GDF15 aligns with previous extensive evidence on the dysregulation of inflammatory pathways following exposure to major traumatic experiences (Baumeister et al. 2016), including within patients with AN (Rossi et al. 2024). Notably, detecting exposure to major childhood traumatic experiences carries significant clinical potential, considering that this specific sub‐population has been characterised as burdened by lower rates of treatment response, higher severity and a higher risk of relapse (Rossi et al. 2024).

In recent years, GDF15 has consistently been described as associated with sarcopenia and cachexia (Kamper et al. 2024), a clinical feature of severity which may also be observed in patients with AN (Tarchi, Cassioli, et al. 2024). Previous meta‐analyses showed GDF15's increases up to *d* = 0.48 in gestational diabetes mellitus (Y.‐C. Lu et al. 2023), *d* = 0.92 in neurodegenerative diseases (Xue et al. 2022), and *d* = 2.91 in patients with lung cancer (Pan et al. 2025). In parallel, studies showed GDF15's increases up to *d* = 0.27 in hyperemesis gravidarum (Fejzo et al. 2019) and *d* = 1.4 for patients with cancer anorexia (Molfino et al. 2020) Current results estimate GDF15's increases in AN to be at the upper limit of these comparisons (*d* = 1.83), indicating strong robust elevations in this clinical condition. Future studies might then explore the potential of GDF15 serum levels to predict morbidity and mortality in patients with AN (Castellini et al. 2023), as well as the diagnostic potential to detect concurrent medical comorbidities.

Although GDF15 did not directly correlate with ED symptoms, our findings suggest that ghrelin mediates the relationship between GDF15 and ED severity (i.e. low BMI in patients with AN). This result provides a preliminary, yet interesting mechanistic explanation for the observed association between high GDF15 and low BMI in these patients. In the authors' interpretation, this mediation effect implies that GDF15 may induce lower BMI through an elevation in ghrelin serum levels. One possible explanation is that chronic energy deprivation in EDs triggers an adaptive hormonal response involving GDF15 elevation, as GDF15 has recently been shown to induce gastric contraction (Garella et al. 2025), and thus possibly ghrelin secretion. This adaptive metabolic response may attempt to modulate feeding behaviour, resulting in decreased energy expenditure, although being also characterised by GDF15‐induced visceral malaise. An adaptive metabolic response following GDF15 elevation may also explain the unsatisfactory response obtained by current GDF15 analogues within clinical trials (Di Santo et al. 2025). In fact, in one phase I trials conducted in overweight and obese individuals, GDF15 analogues induced nausea and vomiting, but were not associated with substantial weight loss (Benichou et al. 2023).

Current results further suggest that GDF15 may exhibit its effects not only by activating central appetite‐regulating neurons, but possibly also peripheral receptors, such as peripheral receptors involved in metabolic response, gastric contractility, insulin sensitivity, mitochondrial function or adipokine secretion (Fichtner et al. 2024; Garella et al. 2025). Following this hypothesis, GDF15 elevations observed in AN may not only represent maladaptive maintaining factors for decreased appetite or visceral malaise (Borner et al. 2020), but also, as previously mentioned, adaptive mechanisms attempting to rescue cellular homoeostasis in light of chronic undernutrition, by modulating peripheral components of metabolic regulation. This adaptive hypothesis for GDF15 may also explain the rapid reversal of cortical thinning observed in AN upon weight restoration (Bernardoni et al. 2016), as GDF15 may promote neuronal survival, prevent oxidative stress and sustain functional recovery in the central nervous system (Isik et al. 2024). Future studies are, however, required to confirm this hypothesis, and explore whether the lack of induction of substantial weight loss by GDF15 analogues is mediated by peripheral mechanisms of action (Di Santo et al. 2025). Future studies are also needed to explore the temporal pattern of association between GDF15, ED symptoms and ghrelin serum levels, possibly employing data retrieved by pharmaco‐modulation, within (randomised controlled) clinical trials.

Current findings have several important implications. From a clinical perspective, measuring GDF15 and ghrelin levels could improve diagnostic accuracy and aid in the identification of patients at higher risk for severe restriction. Additionally, interventions targeting GDF15 and ghrelin interaction may provide novel therapeutic strategies for EDs. For instance, modulating ghrelin activity through GDF15 antagonism could help mitigate the maladaptive metabolic responses seen in EDs (Duncan et al. 2017). From a research standpoint, future studies should explore the longitudinal dynamics of GDF15 and ghrelin in ED patients, assessing whether changes in their levels correspond to treatment response and recovery. This investigation is timely and necessary, considering the lack of effective diagnostic or prognostic biomarkers in EDs (Himmerich and Treasure 2024).

Future translational studies may confirm whether GFRAL expression is restricted to the central nervous system alone (Fichtner et al. 2024). At the present time, the detection of GFRAL in the stomach suggests a morpho‐functional explanation for the association between GDF15 and ghrelin serum levels (Garella et al. 2025). Future experimental studies may thus better describe GFRAL expression in human tissues, and better characterise its interplay with inflammatory cytokines (di Candia et al. 2021). Given the recent description of GDF15 analogues acting as GFRAL ligands (Di Santo et al. 2025), future studies might explore the pharmacological potential of this signalling pathway, possibly employing GDF15 antagonism to rescue GDF15 elevations observed in AN. This line of research could provide deeper insights into the biological basis of EDs in general, and AN in particular, an urgently needed line of research considering the relative lack of pharmacological options for these disorders (Bulik 2021).

### 4.1 Limitations

This study has several limitations that should be considered when interpreting the findings. First, the sample is restricted to individuals of female sex and gender, restricting the potential of present results to inform on clinical populations composed by male or gender‐diverse individuals (Tarchi, Stanghellini, et al. 2024). Future studies should include a more diverse sample to improve the applicability of findings across different sexes and genders. Second, the study only includes participants aged 18 years and older, thereby excluding children and adolescents. Since adolescence is a critical period for the onset of eating disorders (Solmi et al. 2022), future studies should investigate these factors in younger populations. Additionally, the clinical group of patients with ED was mostly representative of patients with AN, potentially limiting the generalisability of reported findings for patients with BN or BED. Similarly, sub‐diagnostic differences (i.e. AN‐r vs. AN‐bp), although of potential interest for future studies, were limited by small sample sizes (38 vs. 22 individuals, respectively), warranting caution in drawing wider conclusions for present results.

Mediation analyses were conducted on cross‐sectional results, which impedes inferring causality and causal direction regarding GDF15's role in ED aetiology or in ghrelin modulation. Future studies are required to substantiate the proposed mechanism of action (i.e. GDF15 inducing ghrelin secretion, possibly through the induction of gastric contractility; Garella et al. 2025), which remains based on associations evaluated in cross‐sectional results and previous animal models, and which may be confounded by further interactions and crosstalk between GDF15 and other signal pathways, as, for instance, inflammatory cytokines.

Healthy control participants were excluded if they reported engaging in compensatory behaviours, such as excessive exercise or purging, even in the absence of a formal eating disorder diagnosis. In fact, previous studies reported that while restricting behaviours might be considered as widely present in the general population, along a continuum of severity (Croll et al. 2002; Tarchi, Merola, et al. 2024; Williamson et al. 2005), binge‐purging and compensatory behaviours are currently considered maladaptive irrespectively of their frequency and severity. However, future studies might focus on populations with sub‐threshold or prodromal symptoms, which are increasingly recognised as clinically relevant (Le Grange and Loeb 2007; McClelland et al. 2020) and which may represent early manifestations of disorders at a higher risk of chronicity and relapse, at least indirectly, as contributors to increased untreated/undiagnosed time (Le Grange and Loeb 2007).

Accumulating evidence shows that individuals with attenuated symptoms can exhibit neurobiological, cognitive, or behavioural alterations similar to those with full‐threshold diagnoses (Johnson et al. 2021; Luo et al. 2024; Wallace et al. 2020). Future studies should therefore prioritise the inclusion of at‐risk populations and individuals with mild symptomatology, in order to evaluate whether, and at which point along the continuum, case‐control differences may arise. Such an approach may yield clinically relevant insights into early identification and intervention strategies.

## 5 Conclusions

This study suggests that GDF15 is elevated in ED patients, while also being positively associated with diagnostic severity. GDF15 was shown to interact with ghrelin to induce lower BMI in patients with AN. These findings provide new insights into the hormonal dysregulation underlying EDs, and highlight the potential of GDF15 as a biomarker for disease severity. Further research is needed to elucidate the causal mechanisms of these interactions and to explore their therapeutic potential in clinical practice.

**Nomenclature**
AN: Anorexia Nervosa
BDI: Beck Depression Inventory II
BED: Binge Eating Disorder
BMI: Body Mass Index
BN: Bulimia Nervosa
DSM: Diagnostic and Statistical Manual of Mental Disorders
ED: Eating Disorders
EDE: Eating Disorder Examination Questionnaire
ELISA: Enzyme‐Linked Immunosorbent Assays
GDF15: Growth Differentiation Factor 15
GDNF: Glial Cell Line‐Derived Neurotrophic Factors
GFRAL: GDNF family receptor alpha like
MIC‐1: Macrophage Inhibitory Cytokine‐1
SCL‐90: Symptom Checklist‐90‐Revised
TGF‐β: transforming growth factor‐β

## Author Contributions

**Livio Tarchi:** conceptualization, data curation, formal analysis, investigation, visualization, writing – original draft. **Giulia Brancolini:** data curation, investigation, methodology, writing – review and editing. **Sara Giachetti:** data curation, investigation, methodology, writing – review and editing. **Gaia Maiolini:** data curation, investigation, methodology, writing – review and editing. **Emanuele Cassioli:** data curation, investigation, methodology, supervision, writing – review and editing. **Eleonora Rossi:** data curation, investigation, methodology, supervision, writing – review and editing. **Gianluca Villa:** conceptualization, supervision, writing – review and editing. **Rachele Garella:** conceptualization, investigation, writing – review and editing. **Roberta Squecco:** conceptualization, supervision, writing – review and editing. **Paolo Rovero:** conceptualization, supervision, writing – review and editing. **Paolo Comeglio:** data curation, investigation, methodology, supervision, writing – review and editing. **Valdo Ricca:** conceptualization, supervision, resources, funding acquisition, writing – review and editing. **Francesco De Logu:** conceptualization, data curation, investigation, methodology, supervision, resources, funding acquisition, writing – original draft, writing – review and editing. **Romina Nassini:** conceptualization, data curation, investigation, methodology, supervision, resources, funding acquisition, writing – original draft, writing – review and editing. **Giovanni Castellini:** conceptualization, data curation, investigation, methodology, supervision, resources, funding acquisition, writing – original draft, writing – review and editing.

## Ethics Statement

All subjects gave their informed consent for inclusion before they participated in the study and provided written consent for the publication of results. The study protocol was approved by the Ethics Committee of the Institution of the promoting centre (IRB codes OSS.14.162 and OSS.24.354, last update to the ethical committee approved in November 2023).

## Consent

All subjects gave their informed consent for inclusion before they participated in the study. All subjects provided written consent for the publication of results.

## Conflicts of Interest

The authors declare no conflicts of interest.

## Data Availability

The data, code and material generated for this study can be shared upon reasonable request to the corresponding author.
